# Editorial: 3D organoid and organ-on-a-chip and their applications for virology and antiviral research

**DOI:** 10.3389/fmicb.2023.1266136

**Published:** 2023-08-08

**Authors:** Yuebang Yin, Lei Xu, Steven Nathaniel Steinway

**Affiliations:** ^1^Department of Gastroenterology and Hepatology, Erasmus University Medical Center, CE Rotterdam, Rotterdam, Netherlands; ^2^National Key Laboratory of Crop Improvement for Stress Tolerance and Production, Shaanxi Key Laboratory of Agricultural and Environmental Microbiology, College of Life Sciences, Northwest A&F University, Xianyang, Shaanxi, China; ^3^Department of Medicine, Johns Hopkins University School of Medicine, Baltimore, MD, United States

**Keywords:** 3D organoid, organ-on-a-chip, virology, antiviral, microphysiology systems

A powerful *in vitro* model plays an essential role in speeding up drug development and the lack of such models could hamper drug discovery for important diseases. Therefore, it is desperately needed to develop a sophisticated *in vitro* model for drug development. The conventional *in vitro* models mainly contain cell lines, primary cells, and *ex vivo* models. However, the limitations of these conventional models limit their applications in drug discovery (Yin et al., 2021). For example, accumulating evidence indicates that cell lines are vulnerable to mutation after culturing for extended periods of time, which might cause inconsistent results between the labs using the same cell line (Liu et al., 2019). The degree of molecular and phenotypic variability across 14 stock HeLa samples from 13 international laboratories was explored, which indicated that a substantial heterogeneity happened between HeLa variants, and the genomic variability has a complex, non-linear effect on transcriptome, proteome, and protein turnover profiles, and prototype patterns explain the varying phenotypic response of different cell lines to *Salmonella* infection (Liu et al., 2019). Many cultured cell lines are demonstrated to be genomically unstable (Muff et al., 2015). On the contrary, primary cell lines are generally considered to be more representative of normal physiology, often undergoing senescence processes and harboring limited potential for self-renewal and differentiation. However, large amounts of cells and cell types are of limited availability, which makes it difficult to broadly utilize isolated primary cells to lead discovery (Zheng et al., 2013). *In vitro* models containing single cell types far from mimick the complexity of *in vivo* organs and tissues which are often composed of multiple cell types (Yin et al., 2019b). Ethical issues and shortage of resources make it difficult to use human tissues as *ex vivo* models.

More recently the development of organoids by Hans Clevers' group has allowed for a *in vitro* culture of organ-like structures containing multiple difference cell types from a renewable stem cell source (Sato et al., 2009). To date, a variety of types of organoids including brain, retinal, kidney, liver, lung, gastrointestinal, cardiac, vascularized, and multi-lineage organoids have been successfully cultured (Yin et al., 2019a). Organoids have been broadly applied in biomedical research, drug discovery, and regenerative medicine (Sato et al., 2009). Organ-on-a-chip model is based on microfluidic devices built using a combination of cell biology, engineering, and biomaterial technology. The microenvironment of the chip mimics that of the organ in terms of tissue interfaces, fluid flow, and mechanical stimulation (Kimura et al., 2018). The advantage of the organ-on-a-chip model is that it could physically and chemically mimic the *in vitro* environment by using microfluidic device technology, maintenance of cellular function, and morphology and replication of organ interactions (Ma et al., 2021). Thus, organoids, organ-on-a-chip, and their combination are promising *in vitro* models in drug discovery.

Emerging studies show that organoids and organ-on-a-chip models can model viral infection and antiviral drugs. For instance, Yin et al. (2015) used primary intestinal organoids to establish a rotavirus infection model, which could be used as an antiviral screening and personalized tool. Han et al. (2021) used lung and colonic organoids to perform a high throughput screen of FDA-approved drugs, and found that several potent inhibitors such as imatinib, mycophenolic acid (MPA), and quinacrine dihydrochloride (QNHC) potently inhibited SARS-CoV-2. In another study, Si et al. (2021) established a microfluidic human-airway-on-a-chip model fabricated by highly differentiated human bronchial-airway epithelium and pulmonary endothelium, which was used for mimicking infections and screening antivirals of influenza A and SARS-CoV-2. Thus, organoids and organ-on-a-chip models hold promising potential to study viral infection, strain-dependent virulence, immunology, novel antivirals, and so on.

The goal of this Research Topic was to explore and obtain elegant and state-of-the-art three-dimensional organoid, organ on a chip and other microphysiology systems (MPS) models, which could be used for studying virus function, host-virus interaction, and antiviral mechanisms/drug targeting (Figure 1). This Research Topic aimed to highlight the most advanced achievements and innovations in 3-dimensional organoid, organ-on a chip and other microphysiology systems (MPS) models and their applications in virology and antiviral research, which should inspire and guide the future direction of the field.

**Figure 1:**
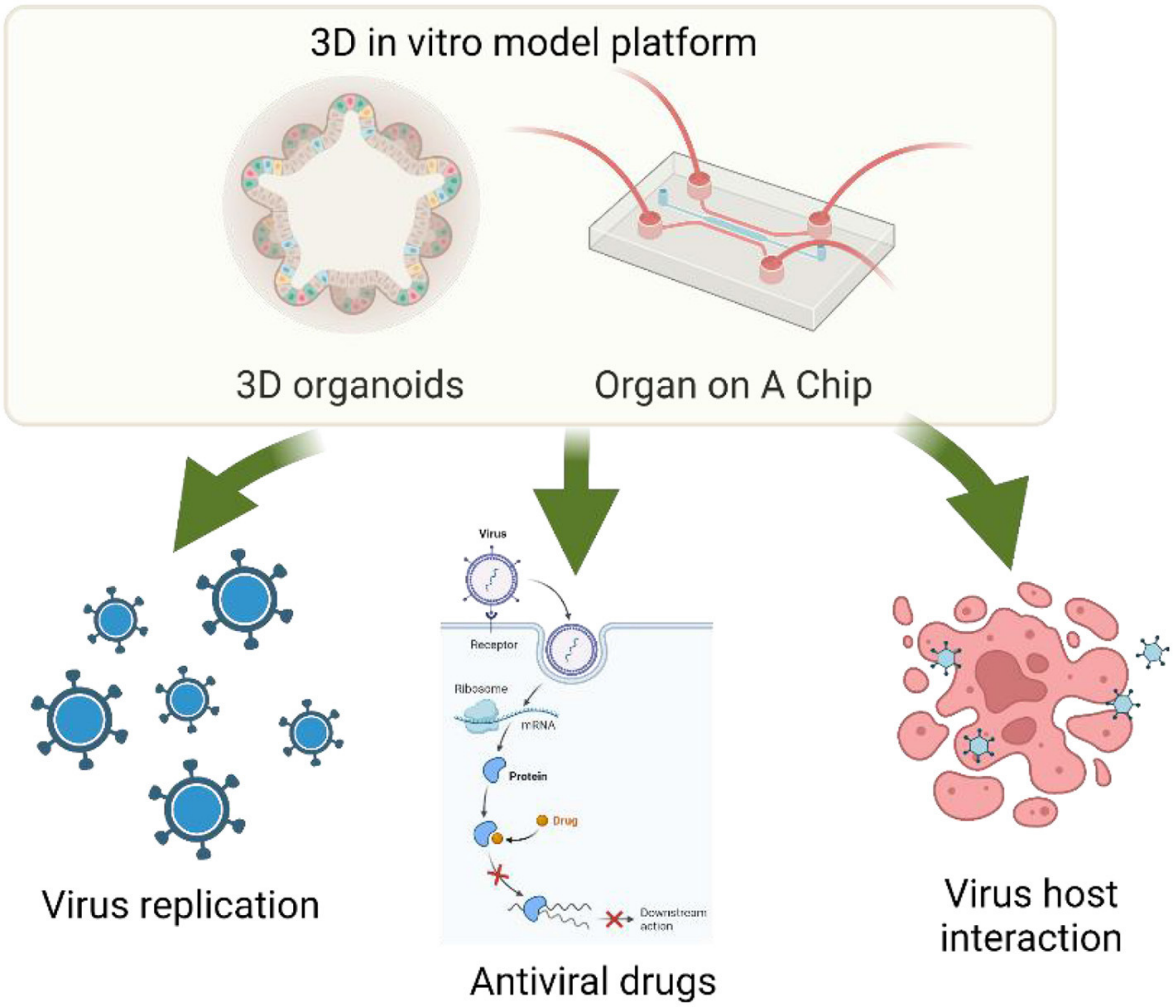
3D organoid and organ-on-a-chip and their applications for virology and antiviral research. Edits by Biorender.

Anjum et al. systematically reviewed current gastrointestinal models of probiotic and pathogen interactions. The review discussed the advantages and limitations posed by a variety of models available to study host-microbe interactions within the gastrointestinal tract that hold the potential to be translated to *in vivo* research. Different models used for studying probiotic and pathogen interactions include traditional non-cellular methods, two-dimensional (2D) models, 3D models, chip-based models, *in vitro* digestive models, *in silico* models, *ex vivo* models, “simpler” animal models, and animal models. 3D intestinal models including enteroids and colonoids were described in detail. organoids can be referred to as “enteroid” when the cells come from the small intestine and “colonoid” when cells are derived from the colon. Intestinal organoids have been used to mimic infections of a variety of pathogens including enterohemorrhagic E. coli, enterotoxin-producing *E. coli*, cholera-toxin, *L. rhamnosus* GG, *Akkermansia muciniphila*, and *Faecalibacterium prausnitzii*. Effects of bacteria (such as *L. reuteri* D8) on epithelial functions were also explored using intestinal organoids.

McDuffie et al. reviewed the applications of physiologically relevant or physiomimetic microsystems as tools for studying viral hepatitis infection in the liver and how the design of these platforms is tailored for the enhanced investigation of the viral lifecycle when compared to conventional 2D cell culture models. Viral hepatitis is a significant contributor to hepatocellular carcinoma and liver disease. However, conventional *in vitro* models are ineffective for studying this transition due to the lack of a functional phenotype of hepatocytes that is permissive to infection long enough to model infection chronically. Importantly, physiomimetic microsystems could help bridge the gap to longer *in vitro* infections by incorporating elements of the hepatic microenvironment to promote the functional longevity of hepatocytes. Physiomimetic models are capable of recapitulating different elements of the hepatocyte microenvironment to sustain an infection-permissive phenotype most notably. Physiomimetic models support co-culture with non-parenchymal cells, 3D morphology, a physiological spatial orientation, and media perfusion. Thus, physiologically relevant or physiomimetic microsystems should be used as a sophisticated model for studying viral hepatitis infection.

Belanger et al. used a saturated transposon insertion mutant pool of *P. aeruginosa* strain PAO1 and transposon insertion sequencing (Tn-Seq), to identify genes conditionally important for survival under conditions mimicking the environment of a nosocomial infection. Importantly, a human skin organoid model was used in the study. Genes involved in nucleotide metabolism, and cobalamin (vitamin B12) biosynthesis, etc., were required for survival of *P. aeruginosa* strain PAO1 *in vivo*- and in host mimicking conditions, but not in nutrient-rich lab medium, Mueller Hinton broth (MHB) was identified in the study. Mutants in genes encoding proteins of nucleotide and cobalamin metabolism pathways were demonstrated to have growth defects under physiologically-relevant media conditions, *in vivo*, and organoid models, and were downregulated in expression under these conditions, when compared to MHB media.

Aknouch et al. proposed an amino acid variation at VP1-145 of enterovirus A71 that determines viral infectivity and receptor usage. Two EV-A71 clinical isolates including C1-91–480 and C1-1185 with either a Q or E at VP1-145, and human fetal intestinal organoids were used in the study. C1-480-Q clinical isolate was found to replicate more efficiently than the C1-1185-E clinical isolate after apical and basolateral inoculation in the human fetal intestinal organoid-derived monolayers, which indicated that VP1-145Q determines increased infectivity of the virus. Site-directed mutagenesis was performed to generate two EV-A71 mutants, with E (VP1-145E) or Q (VP1-145Q) amino acid at VP1-145, which indicated that the EV-A71 VP1-145Q mutant replicated more efficiently after both apical or basolateral inoculation compared to the VP1-145E mutant. Therefore, it was clearly confirmed that the presence of glutamine, as opposed to glutamic acid, at VP1-145 is key for viral infection in a 2D human fetal intestinal model, which was consistent with previous findings in an airway organoid model. Pre-treatment of EV-A71 particles with low molecular weight heparin to block heparan sulfate proteoglycan (HSPG)-binding significantly reduced the infectivity of two clinical EV-A71 isolates (C1-91–480 and C1-1185) and viral mutants carrying glutamine at VP1-145.

Feng et al. proposed a COVID-19 annotation platform named OVIDanno (http://biomedbdc.wchscu.cn/COVIDanno/), which aims to provide a reference resource of intensive functional annotations of differentially expressed genes (DEGs) among different time points of COVID-19 infection in human *in vitro* models. In total, differential expression analysis was executed for 136 individual datasets across 13 tissue types. 4,935 DEGs were identified from the analysis. Multiple bioinformatics/computational biology studies were further performed on these DEGs. Herein, the OVIDanno platform will be a valuable resource for identifying SARS-CoV-2-related genes and understanding their potential functional roles in different time points and multiple tissue types.

In conclusion, the studies in this Research Topic have expanded our understanding of more advanced *in vitro* models including three-dimensional organoid, organ on a chip and other microphysiology systems (MPS) models, especially applications of these models in studying virology and antivirals. The findings of articles of the Research Topics highlight advantages of the exquisite 3D models in virology field.

## Author contributions

YY: Conceptualization, Writing—original draft, Writing—review and editing. LX: Writing—review and editing. SS: Writing—review and editing.
